# Cosmetics: What Do Bruneian Female Adults Believe?

**DOI:** 10.3390/ijerph191710584

**Published:** 2022-08-25

**Authors:** Long Chiau Ming, Nur Hafizah Raudhah Azmi, Hui Poh Goh, Li Ling Chaw, Khang Wen Goh, Nahlah Elkudssiah Ismail, Ganesh Sritheran Paneerselvam, Andi Hermansyah

**Affiliations:** 1 Pengiran Anak Puteri Rashidah Sa’adatul Bolkiah Institute of Health Sciences, Universiti Brunei Darussalam, Gadong BE-1410, Brunei; 2 Department of Pharmacy Practice, Faculty of Pharmacy, Universitas Airlangga, Surabaya 60115, Indonesia; 3 Faculty of Data Science and Information Technology, INTI International University, Nilai 71800, Malaysia; 4 Malaysian Academy of Pharmacy, Puchong 47160, Malaysia; 5 School of Pharmacy, Faculty of Health and Medical Science, Taylor’s University, Subang Jaya 47500, Malaysia

**Keywords:** gender equality, public health, healthcare access, cosmetology, sustainability and community

## Abstract

Objectives: The study aimed to measure the level of attitudes and the current practices of the female community in Brunei Darussalam regarding the usage of cosmetics. Methods: An online survey was conducted using a non-probabilistic snowball sampling approach via the social media channels WhatsApp and Instagram. The inclusion criteria were female Bruneian citizens or permanent residents, aged between 18 and 65 years old, who can understand English or Malay, and use cosmetic products at least once a day. Results: A total of 445 participants responded to the online survey. Most of the participants agreed that the use of cosmetic products improves one’s physical appearance to the public (391, 87.8%) and also improves self-confidence (405, 91.1%). There were significant differences in monthly cosmetic product expenses and participants’ attitudes about safe cosmetic use (*p* = 0.001). No significant changes in the individuals’ attitudes based on their age or educational level were observed. Overall, the participants had a good level of cosmetic safety practice. Almost half of the participants use social media to obtain information regarding what cosmetics to use or purchase. Conclusion: There is a medium to high level of attitude and a high level of practice regarding the safe use of cosmetics among Bruneian female adults. Social media was the main source of information for the respondents, followed by friend circle and family members.

## 1. Introduction

According to the FDA, a cosmetic is defined as “a product (excluding pure soap) intended to be applied to the human body for cleansing, beautifying, promoting attractiveness, or altering the appearance”. Shampoos, hair dyes, toothpaste, and deodorants are all considered personal care goods, in addition to lipsticks, and eye and facial makeup [1]. An online survey of 1039 adult female and male participants in the United States showed that 82% of female respondents used skincare products at least once a week, while 64% of male respondents said the same [2]. Moreover, there was an increase in the worldwide cosmetics markets growth rate from 4.9% in 2018 to 5.5% in 2019 [3]. According to a study conducted in Saudi Arabia, where 50.6% of the 425 participants reported having at least one adverse event with a cosmetic product in the previous two years, the number of cosmetic products used per day has a statistically significant relationship with the occurrence of cosmetics-related adverse events; using more cosmetic products each day increases the likelihood of having adverse events [4].

In some countries, cosmetovigilance is practiced, with the goal of monitoring adverse reactions to cosmetic products [5]. Cosmetovigilance, according to Vigan and Castelain (2014) [5], is a type of public health monitoring that collects information on the safety of cosmetics and their ingredients. Furthermore, the level of attitudes and practices of the community in some countries regarding the safe use of cosmetic products are also low [6,7,8]. There are some examples of adulterated ingredients in cosmetic products that were illegally marketed in Brunei Darussalam and have been banned by the government, such as hydroquinone and steroids [9]. The reported adulterated cosmetics were banned from being imported and sold in Brunei Darussalam [10]. Due to a rise in cosmetics manufacture and importation, Brunei formalised the regulation of cosmetic products through the implementation of the Medicine (Cosmetic Products) Regulation in 2007, in line with the ASEAN (Association of Southeast Asian Nations) Cosmetic Directive (ACD). Prior to importing cosmetic products into Brunei Darussalam, manufacturers or importers must notify the Ministry of Health [9]. Those involved in the retail of these products, including online retail, are reminded that importing and marketing cosmetic products in the local market without a Cosmetic Product Notification Acknowledgement Letter issued by the health authority is illegal under the Medicines (Cosmetic Products) Regulation, 2007. A conviction for violating these restrictions carries a fine of up to BND 5000 (equivalent to USD 3595), a sentence of up to two years in prison, or both [11].

To our knowledge, there is currently no information available about the community’s attitudes and practices around cosmetics in Brunei Darussalam. Hence, we conducted an online survey to determine the attitudes and identify the current practices towards the safe use of cosmetics among female adults in the country. Findings from this study may increase community awareness of the side effects of using cosmetic products. The findings can serve as a baseline of the knowledge level on the safety of cosmetics.

## 2. Methods

### 2.1. Study Design

This online survey was conducted using a non-probabilistic snowball sampling approach, whereby the survey link was shared to known contacts on social media platforms such as WhatsApp and Instagram, who in turn were encouraged to share the link with their friends and relatives meeting the inclusion criteria. The inclusion criteria were female Bruneian citizens or permanent residents, aged between 18 and 65 years old, able to understand English or Malay, and using cosmetic products at least once a day. The definition of a cosmetic applied in this study was based on the aforementioned definition by the FDA in the United States [1].

### 2.2. Study Instrument

The questionnaire for the survey was adopted and modified from a published study [6]. It was distributed in both English and Malay, the latter being the official language of Brunei, though the majority of the population have a good command of English too. Pre-testing was conducted with 25 female participants to ensure that the questions were comprehensible to the target audience and relevant to the setting, and to assess the questions’ reliability using Cronbach’s alpha test.

The finalised questionnaire consisted of a total of 36 questions comprising 4 sections: (1) General socio-demographic characteristics; (2) Attitudes towards cosmetic products; (3) Practices towards cosmetic products; and (4) Sources of information. For the first section, participants’ ages were collected as categorical variables (18–35 and 36–65 years old). Participants’ education levels were also collected as categorical variables. Secondary education level comprises those with O-level, A-level, or International Baccalaureate qualifications; post-secondary non-tertiary education level includes those with Foundation, Certificate, or Diploma qualifications; and tertiary education level includes those with Bachelor’s, Master’s, or PhD qualifications. Responses for attitudes were collected using a 5-point Likert scale (strongly disagree = 1, disagree = 2, neutral = 3, agree = 4, and strongly agree = 5), while responses for practices (no, unsure, and yes) and sources of information were collected in a descriptive manner. The attitude and practice sections had Cronbach’s alpha values of 0.84 and 0.69, respectively, indicating an acceptable internal consistency [12].

### 2.3. Data Analysis

All questionnaire responses were collected using Google Forms and uploaded for analysis as a Microsoft Excel spreadsheet. Descriptive statistics (percentages, mean, and standard deviation) were performed. The total attitude score (40 points) was then divided into 3 categories: low (total attitude score of ≤15), medium (total attitude score from 20 to 30), and high (total attitude score from 31 to 40). Associations between demographic characteristics and total attitude scores were investigated using the Chi-square test. A *p*-value of <0.05 was considered as statistically significant. All analyses were conducted using Microsoft Excel and R (ver. 4.1) [13].

### 2.4. Ethical Approval

The Joint Research Ethics Committee of the PAPRSB Institute of Health Sciences provided full approval of the study, which was given the reference number UBD/PAPRSBIHSREC/2021/74.

## 3. Results

A total of 445 participants were included in this study (Table 1), with the majority of them between 18 and 35 years old (328, 73.7%). A significant proportion of the participants were working (277, 62.2%), had a monthly income of less than BND 1000 (229, 51.5%), and spent more than BND 100 per month on cosmetic products (354, 79.6%).

### 3.1. Attitudes of the Community towards the Safe Use of Cosmetics

Attitudes towards safe use of cosmetics were generally high among our study participants, with a mean of overall total attitude scores ranging between 2.9 and 4.5 (Table 2). A high percentage of them agreed or strongly agreed that the use of cosmetics can cause allergies (231, 51.9%), can improve one’s physical appearance (391, 87.8%), and can improve self-confidence (405, 91.1%). More than half of the participants (289, 54.9%) agreed or strongly agreed that before the cosmetics are sold on the market they must be well tested in laboratories. Only 19.3% (*n* = 86) agreed that using cosmetics can cause reproductive health problems.

Overall, the study participants had medium to high attitudes towards the safe use of cosmetics (Table 3). Age groups, education levels, and monthly expenses for cosmetic products were the three chosen variables for the study, as each group had a balanced number of people. None were classified with a low attitude score. Notably, 62.8% of participants aged from 18 to 35 years old had medium attitude scores towards the safe use of cosmetics. No statistically significant differences were observed between total attitude scores and age group (*p* = 0.291), or between total attitude scores and education level (*p* = 696). Those who spent less than BND 100 per month on cosmetic products were more likely to have medium to high total attitude scores when compared with those spending more than BND 100 (*p* = 0.001).

### 3.2. Practices of the Community towards the Safe Use of Cosmetics

Table 4 shows the responses of the participants regarding practices for the safe use of cosmetics. Most participants wash their hands before applying cosmetic products to their face or body (404, 90.8%), read the labels of the cosmetic products (353, 79.3%), and share information about the hazards of using cosmetics (300, 67.4%). Less than half (42.7%) reported buying skin or lip products with Vitamin A.

### 3.3. Participants’ Source Information Regarding What Cosmetics to Buy or Use

**Figure 1:**
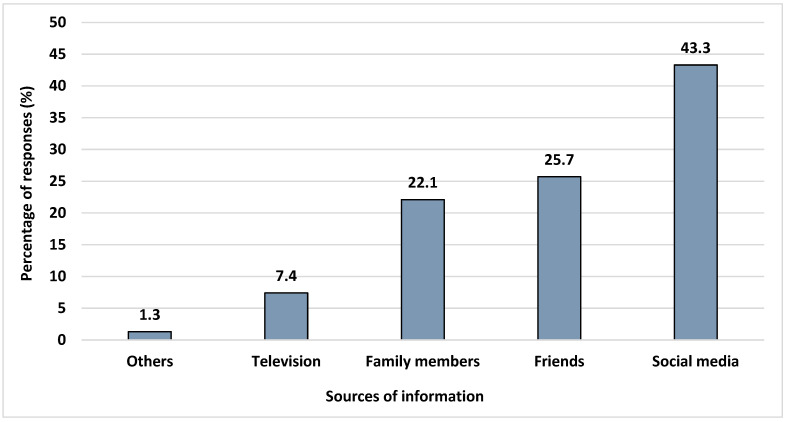
Percentage of responses on the source of information regarding what cosmetics to use or buy (*n* = 445).

Study participants tend to rely on a variety of sources for information about which cosmetics to buy or use, such as family members (22.1%), friends (25.7%), social media (43.3%), television (7.4%), and others (1.3%, Figure 1). Nearly half of the participants reported that social networking sites provided them with knowledge about what cosmetics to buy and use. Other sources reported include counselling from a beautician or aesthetician, shop posters, pharmacy store posters, and magazine advertisements.

## 4. Discussion

This is the first study of its kind in Brunei Darussalam to examine the general public’s attitudes and practices regarding the safe use of cosmetics. Brunei Darussalam began regulating cosmetic products in 2007 under the Medicine (Cosmetic Products) Regulation, in line with the ASEAN Cosmetic Directive (ACD). There were significant number of participants who stated that the use of cosmetics can improve one’s physical appearance (391 participants) and self-confidence (405 participants). The findings of the study are similar to a study performed in Malaysia during the COVID-19 pandemic [14], which found that the application of cosmetics was aimed to further the appeal of one’s appearance, especially during a time when online conferencing was widely used. This is the primary motive for consumers to purchase cosmetic items: because they want to improve their existing appearance and correct features that they dislike [15]. Moreover, applying cosmetics stimulates the senses of touch, smell, and sight, which boosts one’s physiological pleasure and confidence level [16]. This is also in line with the findings of Handriana et al. (2020), who found that some women were unaware of their unsatisfactory face traits until they came across cosmetic products designed to correct them. They also noted that there is presently a product for every aspect of the face, and that as innovation and technology advance, the products will become increasingly invasive as they transition from temporary to permanent characteristics [17].

Based on the findings, there was a significant difference between monthly expenses on cosmetic products and the attitude of the participants (*p* = 0.001). This is also supported by Mohammed et al. (2021), who found a significant relationship between monthly cosmetic product expenditures and participants’ attitudes in Malaysia [14]. The monthly expenses for cosmetic products served as a surrogate value of the consumers’ particular interest in terms of quantum of time and money invested on cosmetics, as indicated by its statistical significance. The results show that the Bruneian community has a medium to high level of attitude towards the safe use of cosmetics. These include notions such as augmenting physical appearance to boost the users’ self-confidence. Similarly, a study performed among the women at Najran University in Saudi Arabia revealed that there was an adequate attitude level in the area of cosmetics [18]. As shown in Table 2, more than half of the participants believe that using cosmetics could cause cancer. There are numerous controversies surrounding cosmetic products and increased cancer risk, though there is still a lack of scientific evidence to prove the relationship [19,20]. Similarly, a study on the everyday use of cosmetics and increased risk of breast cancer shows that there is no sufficient epidemiological data to support the relationship [21].

Furthermore, it was found that a large number of participants use sunscreen with SPF 50 and above. This is consistent with a study carried out in Yazd City, Iran, in which a high number of participants agreed that sunscreen should be used at an early age [8]. The usage of sunscreen has been shown to reduce the detrimental effects of UV radiation on the skin [22]. However, this contradicts a study conducted in Saudi Arabia, which indicated that there was an equal proportion of people who used and did not use sunscreen [7].

Additionally, a large proportion of participants purchase skin or lip products containing Vitamin A. One study showed that after 12 weeks of use, the retinol formulation significantly reduced fine wrinkles [23]. This also aligns with a study conducted among journalists in the Philippines in 2015, which showed that the respondents bought cosmetic products containing Vitamin A, such as retinol, retinyl palmitate, and retinyl acetate [6].

The safety of cosmetics has emerged as an important concern for every cosmetics user. Based on our results, participants are particularly concerned about moisturisers that contain petroleum jelly, want skin care products containing Vitamin A and, most importantly, paraben-free products. The knowledge on “paraben-free” was built on the tagline of cosmetic advertisement whereby the outer product label would clearly specify that. This is because the use of paraben-containing products could lead to adverse health outcomes such as estrogenic effects, disrupting hormones in the body, causing anguish and damage to fertility and the reproductive organs, as well as affecting birth outcomes [24]. While the use of petroleum-containing cosmetics is generally safe, provided it is properly refined, incompletely refined petrolatum could potentially be contaminated with polycyclic aromatic hydrocarbons, which cause cancer [25].

The use of Vitamin A-containing cosmetics has proven to reduce the appearance of wrinkles and improve skin elasticity [26]. Furthermore, some respondents chose to select products with a sun protection factor (SPF) 50 and above. Simplistically, most of the public believes that applying sunscreen with SFP 50 and above is best, but scientifically, SPF 50 gives only 1% more protection against UV rays (98%) than SPF 30 (97%) [19].

According to the findings, there is a high level of safe practice around cosmetics use, particularly when it comes to whether or not participants wash their hands before applying cosmetic products to their face or body. For example, 90.8% of the respondents agreed that this affects the safety of using cosmetics. This is consistent with the findings of Najran University in Saudi Arabia, which indicates that there are satisfactory practices in this regard [18].

Information regarding cosmetics can now be easily accessed via social media in this modern era of globalisation. This was shown by the findings of this research, which revealed that nearly half of the people surveyed obtain their knowledge about what cosmetics to buy or use from internet sites such as TikTok, Google, YouTube, and Instagram. They also obtain their knowledge from relatives and friends. This was different from the findings of Serrano (2015), who indicated that the participants received their information regarding cosmetics from mass media such as newspapers and magazines. As there are numerous sources to acquire information on cosmetic products, the public should be educated on the reliable sources. In addition to the FDA website, all countries have their own governmental agencies which control and regulate the safe use of cosmetic products, such as Control of Cosmetic Products in Malaysia, and the Korea Cosmetics Association. Consumers can also search for the information related to their cosmetic products on these authentic websites [27,28].

The goal of this study was to explore the public’s attitudes and practices regarding the safe use of cosmetics. We can examine and understand the public’s viewpoints on the definition and the safe use of cosmetics as a result of the study’s findings. In addition, this study encourages the continuation of cosmetovigilance, which is useful for the safe monitoring of cosmetics usage. Through this, any adverse reaction from the use of cosmetics can be detected and prevented. Therefore, consideration should be given to further strengthening the idea of cosmetovigilance among consumers, vendors, and other stakeholders.

### Strengths and Limitations of the Study

The study comprised a large sample size, with a balanced representation of people of each age and educational category level. However, the study was only open to female participants, as colloquially, cosmetics are directly translated to makeup products catering only for females. Future studies should cater specifically to males by introducing the term cosmetics as skin cleansing and hygiene products, to change the myth that cosmetics are only for women. Another limitation of this study was that the questionnaire was only distributed via Instagram and WhatsApp. Only followers and contacts were contacted for the survey. People who do not use these social media platforms, such as those who primarily use TikTok or Facebook in their daily lives, were not reached.

## 5. Conclusions

The female communities in Brunei Darussalam generally have a medium to a high degree of attitude and a high level of practice when it comes to the safe use of cosmetics. It is noteworthy that there was a significant difference between monthly expenses on cosmetic products and the attitude of the participants, which denotes that females that are willing to invest more money towards their appearance tend to portray better self-confidence. Raising public knowledge on the safe use of cosmetics is necessary to reduce the number of reports of cosmetic side effects. Aside from this, more in-depth research into community attitudes and practices regarding the safe use of cosmetics in Brunei Darussalam should be conducted; more questions could be added, because there is still insufficient research on the topic, particularly on public knowledge.

## Figures and Tables

**Table 1:** Demographic characteristics of study sample (*n* = 445).

Variable	*n* (%)
**Age group (in years)**	
18–35	328 (73.7)
36–65	117 (26.2)
**Ethnicity**	
Bruneian Malay	430 (96.6)
Bruneian Chinese	13 (2.9)
Others	2 (0.4)
**Marital Status**	
Single	225 (50.6)
Married	220 (49.4)
**Education level**	
Secondary	108 (24.3)
Post-Secondary Non-tertiary	133 (29.9)
Tertiary	204 (45.8)
**Occupation**	
Student	102 (22.9)
Working	277 (62.2)
Unemployed	66 (14.8)
**Monthly income**	
Less than BND 1000	229 (51.5)
BND 1000–BND 2000	87 (19.6)
BND 2001–BND 3000	64 (14.4)
More than BND 3000	65 (14.6)
**Monthly expenses for cosmetic products**	
Less than BND 100	91 (20.4)
More than BND 100	354 (79.6)

Note: BND 1 is equivalent to USD 0.72 (currency rate 25 June 2022).

**Table 2:** Attitudes on the safe use of cosmetics (*n* = 445).

Attitudes Statement	1	2	3	4	5	Mean (SD)
	*n* (%)	*n* (%)	*n* (%)	*n* (%)	*n* (%)	
The use of cosmetics can cause allergies	12 (2.7)	25 (5.6)	177 (39.8)	130 (29.2)	101 (22.7)	3.6 (0.98)
The use of cosmetics improves one’s physical appearance to the public	2 (0.4)	11 (2.5)	41 (9.2)	191 (42.9)	200 (44.9)	4.3 (0.77)
The use of cosmetics improves one’s self-confidence	0 (0)	5 (1.1)	35 (7.9)	148 (33.3)	257 (57.8)	4.5 (0.69)
The use of cosmetics can cause cancer	39 (8.8)	79 (17.8)	224 (50.3)	81 (18.2)	22 (4.9)	2.9 (0.95)
Cosmetics are integral to our lives	15 (3.4)	38 (8.5)	186 (41.8)	141 (31.7)	65 (14.6)	3.5 (0.96)
The use of cosmetics can cause reproductive health problems	45 (10.1)	97 (21.8)	217 (48.8)	67 (15.1)	19 (4.3)	2.8 (0.95)
Cosmetics are well-tested in the laboratories before they are sold	2 (0.4)	32 (7.2)	122 (27.4)	127 (28.5)	162 (36.4)	3.9 (0.98)
Expiration dates are very clear in the packaging	10 (2.2)	45 (10.1)	74 (16.6)	106 (23.8)	210 (47.2)	4.0 (1.12)

Scale: 1—strongly disagree; 2—disagree; 3—neutral; 4—agree; 5—strongly agree.

**Table 3:** Association of socio-demographic characteristics with the level of attitude score (*n* = 445).

Characteristics	Total Attitude Score			*X^2^-Statistics ^a^* *(df)*	*p* Value
	Low ^1^	Medium ^2^	High ^3^		
	*n* (%)	*n* (%)	*n* (%)		
**Age group (in years)**					
18–35	0 (0)	206 (62.8)	122 (37.2)	1.12 (1)	0.291 ^a^
36–65	0 (0)	67 (57.3)	50 (42.7)		
**Education level**					
Secondary	0 (0)	70 (64.8)	38 (35.2)	0.72 (2)	0.696 ^a^
Post-Secondary Non-Tertiary	0 (0)	80 (60.2)	53 (39.9)		
Tertiary	0 (0)	123 (60.3)	81 (39.7)		
**Monthly expenses for cosmetic products**					
Less than BND 100	0 (0)	231 (65.3)	123 (34.8)	11.14 (1)	0.001^a,^*
More than BND 100	0 (0)	42 (46.2)	49 (53.9)		

^a^ Chi-square test for independence; * Statistically significant *p* value; ^1^ Low ≥ 15 total attitude score; ^2^ Medium = total attitude score from 16 to 30; ^3^ High = total attitude score from 31 to 40 (maximum score: 40).

**Table 4:** Responses of participants regarding the practices of the safe use of cosmetics (*n* = 445).

Practices Statement	No	Unsure	Yes
	*n* (%)	*n* (%)	*n* (%)
I will share information about the cosmetic hazards (e.g., of hazard: spreading bacteria on the skin. Irritation and scratches on the eye. Fire hazards, in the case of aerosol products such as hairspray. Allergic reactions or sensitivity to ingredients)	19 (4.3)	126 (28.3)	300 (67.4)
I read the labels of cosmetic products	19 (4.3)	73 (16.4)	353 (79.3)
I buy skin/lip products with Vitamin A (e.g., products that contain retinol: anti-aging)	75 (16.9)	180 (40.4)	190 (42.7)
I buy products labelled ‘paraben-free’	31 (7.0)	164 (36.9)	250 (56.2)
I buy cosmetics that claim to have medicinal value	47 (10.6)	184 (41.3)	214 (48.1)
I consult guides for safer cosmetics	42 (9.4)	92 (20.7)	311 (69.9)
I use petroleum jelly (e.g., Vaseline)	141 (31.7)	51 (11.5)	253 (56.9)
If I have young children, I would use baby powder on them	126 (28.3)	127 (28.5)	192 (43.1)
I use sunscreen with SPF 50 and above	76 (17.1)	86 (19.3)	283 (63.6)
I wash my hands before applying cosmetics to my face or body	6 (1.3)	35 (7.9)	404 (90.8)
